# Supplementary material for: Optimizing heat pump unit selection for recirculating aquaculture workshops through computational fluid dynamics
Source: PLoS One. 2024 Jul 10;19(7):e0299997. doi: 10.1371/journal.pone.0299997 (PMC11236187; doi:10.1371/journal.pone.0299997)
Supplement: Attachment — Submitted filename: Report_report_PONE-D-23-39332.pdf [file pone.0299997.s005.pdf]

PLOS ONE

Manuscript ID: PONE-D-23-39332
Selection of Heat Pump Units for Recirculating Aquaculture Workshops Using Computational Fluid Dynamics: Numerical Simulation

The selection of water temperature regulation equipment plays a crucial role in the design of workshops. At present, the choice of water temperature control equipment is usually based on the volume of the fish pond and thermal parameters calculation, combined with aquaculture experience. Due to limitations such as the environment, climate, and types of fish, empirical formulas are only applicable under specific conditions, lacking universal reference value. With the popularization of computational fluid dynamics (CFD) calculation methods, this paper proposes to apply CFD simulation of the temperature field to accurately calculate the heat exchange value between indoor air and water, providing a new approach for equipment selection**. I have made some comments as follows:**

Major issues:

1. The novelty of this study needs to be clarified. Please describe this adequately.
2. The validation part needs to be clarified. In Figure 2, it is unclear how to get the experimental data.
3. The description of the experimental part is not elaborately described.
4. The section and subsection fronts need to be distinguished.
5. Provide proper reference for formulas 1 and 2.
6. Describe elaborately how the simulation results help control water temperature equipment. They need to be more apparent to the audience.
7. Where is the phase change mode working, and what is the interfacial condition for the phase change?
8. What is the flow governing parameters? If it is Reynolds number, then describe how to influence the Re of the flow and temperature pattern.
9. Is it force convection or mixed convection? Describe clearly.
10. The description of the CFD part needs to be better. The CFD study needs to be clarified here. The authors should elaborate on this and correlate the necessity of this CFD study.

Minor Issues:

Page 1

Abstract

Line 15: : “parameters” should be corrected as “**paramerer**”.

Line 25: “There should be a **a** before 12th word “reference”.

Page 1-3

1. Introduction

Line 39: “There should be a **space** after 4th word “methods”.

Line 41:“There should be replace and with comma.No need to repetation of and after 6th word “motion” we can write "numerical methods to simulate fluid motion, heat, and mass transfer processes.".

Line 42: “in**”** should be corrected as “for”.

Line 45: “There should be a **and** after 11th word “temperature”.

Line 46: “There should be a **The** after 9th word “improving”.

Line 73: “There should be a **The** before 9th word “CFD”.

Line 73: “There should be a **The** after 8th word "'and'.

Line 118: “material**”** should be corrected as “materials”.

Page 4-9

2. Establishment and Solution of the CFD Model for Factory-Based Recirculating Aquaculture Workshops

Line 159: “There should no be **Comma** after 10th word "indoors".

Line 221: “There no need to repeat the word considers 7th word "also".

Line 268: “are**”** should be corrected as “is” after the 5th word.

Line 268: “There should be a **a** after 8th word "as".

Line 268: “There should be a **Hyphen** between "third" and "type" after 8th word "as". .

Line 271: “There should be a **Hyphen** between "first" and "type" after 10th word "as".

Line 272: “bottom**”** should be corrected as “bottoms” after the 5th word.

Line 288: “There should be a **The** before 9th word “Zhuanghe”.

Line 298: “There should be **Of The** before 6th word “water”.

Page – 10

3. Experimental Design and Model Validation for a Factoryy-based Recirculating Aquaculture Workshop

Line 354: “There should be **The** before 3rd word “summer”.

Page – 11-16

4. Equipment Selection for Recirculating Aquaculture Workshop

Line 379: “There should be **for** after 5th word “allows”.

Line 458: “There should no be **Comma** after 3rd word "unit".

Line 453: “There should be **the** after 6th word “in”.

Page- 17

5. Conclusion

Line 472: “was**”** should be corrected as “were” after the 4th word "results".
