# Supplementary material for: Optimizing heat pump unit selection for recirculating aquaculture workshops through computational fluid dynamics
Source: PLoS One. 2024 Jul 10;19(7):e0299997. doi: 10.1371/journal.pone.0299997 (PMC11236187; doi:10.1371/journal.pone.0299997)
Supplement: Attachment — Submitted filename: reply to comments.docx [file pone.0299997.s006.docx]

**1 In the abstract, the authors must avoid general description and focusing on the methodology, the results, and conclusions.**

Reply：Simplify the sentence “Due to limitations such as the environment, climate, and types of fish, empirical formulas are only applicable under specific conditions, lacking universal reference value. ”in the abstract，And deleted the sentence "With the popularization of computational fluid dynamics (CFD) calculation methods"

And the model results are added as follows:

It's on line 23： establishing a 3D unsteady-state Computational Fluid Dynamics model. The model considers outdoor temperature, solar radiation, and phase-change heat transfer in water. Comparison with experimental data reveals a root mean square error of 0.45°C for the simulated results. During summer, the highest cooling load occurs at 16:00, reaching 94.6 kW. It is recommended to employ the Daikin GCHP-40MAH ground source heat pump as the water temperature control equipment. CFD simulation validates its effectiveness in shaping the indoor temperature field post-installation.

**2 In section 1. Introduction, the authors spent more than on page talking about the well-known information about CFD role for solving heat transfer model. This introduction must be reduced and the introduction should focus on the main paper objective. And more references must be added about the objective of the paper. Moreover, nothing mentioned about the CFD software that has been used by the authors.**

Reply：I have condensed the section discussing the role of CFD in solving thermal models and provided a brief introduction to ANSYS, outlining the reasons for choosing ANSYS Fluent.

It's on line 62：CFD plays a crucial role in accurately selecting heat pumps for recirculating aquaculture systems, providing precise heat load calculations tailored to diverse building characteristics and geographical environments. This significantly reduces the investment in water temperature control equipment.ANSYS is a finite element analysis software developed by the American company ANSYS in 1970, integrating capabilities for structural, fluid, electrical, and magnetic field analysis. Widely employed across various industries, it is particularly prevalent in simulating temperature fields in greenhouse environments for vegetable cultivation and has gradually found applications in temperature field simulations for aquaculture workshops in recent years. In summary, this paper utilizes Ansys Fluent as the tool for simulating the temperature field within the workshop, aiming to predict the cooling load and facilitate equipment selection.

More relevant references have been added

It's on line 81：Shenghan Zhou and colleagues conducted a study using FLUENT software to simulate the temperature field in a plant factory. Their research focused on the detailed three-dimensional modeling of temperature distribution influenced by LED light heat dissipation and boundary conditions, emphasizing the significance of accurate modeling in such environments[1]. Long Yang and his team utilized FLUENT in their research to simulate the air flow and temperature fields in a gosling house with vertical wall attached jet ventilation in a cold region. This study investigated the effects of different air supply velocities on the thermal environment, providing essential insights for managing ventilation in livestock housing under cold climate conditions[2]. Cruz Ernesto Aguilar Rodriguez and collaborators applied FLUENT software in their research for simulating heat and mass transfer processes in greenhouses. Their work highlighted the importance of heating systems like pipe heating and its impact on crop yields, energy consumption, and operational costs[3]. The study employed computational fluid dynamics to analyze factors such as air movement, temperature gradients, and crop transpiration, crucial for understanding the dynamics of greenhouse systems. The aforementioned studies demonstrate that CFD has been widely applied in the agricultural field, and the use of Fluent software for temperature field simulations is quite mature.

**3 In section 2.1, a schematic diagram for the physical model must be added to demonstrate the details that were mentioned in this section.**

Reply：Specific architectural photos have been added to illustrate the details mentioned in that section

It's on line 153：

**Fig. 1** Architectural diagram of the recirculating aquaculture workshop

**1.4 In section 2.2 Computational Domain and Grid Division, lines 175-179, the choosing of scheme 2 must be justified by drawing a graph between the values of highest temperature of the indoor air and the number of the Grids for each scheme.**

Reply：You've already added a "Grid Independence Line Chart" in the document to demonstrate that the grid partitioning in Scheme 2 is sufficient for calculating the accuracy requirements.

It's on line 191

**1.5 It preferable to present a cross-section for the Grid mesh of Fig.1.**

Reply：You have modified Figure 1 to a cross-sectional diagram for a better description of the effectiveness of grid partitioning.

It's on line 181

**1.6** **Add reference(s) for the equations 1-2.**

Reply：I have already added reference(s) for equations 1-2

It's on line 200： Reference is “Wang Fujun. Computational Fluid Dynamics Analysis—Principles and Applications of CFD Software [M]. Beijing. Tsinghua University Press. 2004”

**1.7 In section 2.3 Basic Control Equations, lines-206-207, how did the authors decide that the flow is laminar? Justify the assumption by calculating Rayleigh number.**

Reply：Added a description of Reynolds number calculation.

It's on line 220：The fluid flow is slow, as measured by a hot-wire anemometer, indicating a near-wall air velocity of 0.01 m/s. Using the height of the workshop as the characteristic length, the calculated Reynolds number is 2000, which falls within the laminar flow regime.

**1.8 The range of application of Equation 3.**

Reply：In front of Formula 3, the application scope has been added.

It's on line 268：One of the empirical formulas is particularly suitable for scenarios where the flow speed is less than 0.5 m/s, aligning well with the parameters of this study

**1.9 In section 2.5 Computational Domain and Boundary Conditions, The author must add the mathematical formulation of the boundary conditions mentioned in this section.**

Reply：The function used in the boundary conditions is the result of fitting the formula of a third-order Fourier function using MATLAB. Due to the lengthy nature of this function, it is not explicitly presented in the document. Below is the function used for the fitting

clear all;

close all;

clc;

data = importdata('zhuanghe-hour.dat'); % Load data

tem = data.data(4922:4944,6); % Temperature data

nt = length(tem); % Number of data points

ts = 1:3600:3600*(nt-1)+1;

n = 8; % Order of trigonometric fitting

T = max(ts) - min(ts);

omega = 2 * pi / T; % Calculate angular velocity

% Calculate coefficients of trigonometric functions

a0 = 2 * trapz(ts, tem) / T;

for i = 1:n

c{i} = diag(tem * cos(i * omega * ts));

a(i) = 2 * trapz(ts, c{i}) / T;

s{i} = diag(tem * sin(i * omega * ts));

b(i) = 2 * trapz(ts, s{i}) / T;

end

%% Function form

syms t w

f = a0 / 2;

for i = 1:n

f = f + a(i) * cos(i * w * t) + b(i) * sin(i * w * t);

end

vpa(f, 4) % Display function

disp(['Where w = ', num2str(omega)])

%% Calculate Root Mean Square Error

ft = zeros(size(ts));

for i = 1:n

ft = ft + a(i) * cos(i * omega * ts) + b(i) * sin(i * omega * ts);

end

ft = ft + a0 / 2;

RMse = sqrt(sum((ft' - tem).^2) / nt);

disp(['Root Mean Square Error is ', num2str(RMse)]);

%% Calculate temperature at other time points for plotting

t2 = 1:10:3600*(nt-1)+1;

ft2 = zeros(size(t2));

for i = 1:n

ft2 = ft2 + a(i) * cos(i * omega * t2) + b(i) * sin(i * omega * t2);

end

ft2 = ft2 + a0 / 2;

figure;

scatter(ts, tem, '.'); % Plot original data points

hold on;

plot(t2, ft2, 'LineWidth', 1); % Plot predicted data

hold off;

xlabel('Time (s)');

ylabel('Temperature');

legend('Experimental Data', 'Fitted Function');

**1.10 The details of the equation 5. Where did the authors get this equation?**

Reply：Equation 5 is the result of fitting the above-mentioned function to the outdoor temperature values on October 20th

**1.11 In section 42, The Figure 5 should be Figure 6.**

Reply：

**Fig. 8** The temperature distribution of section X-axis of the workshop at 12:00

**1.12 In section 4.2, a streamlines of velocity distribution.**

Reply：Results and appropriate descriptions of the velocity field have been added to the article.

It's on line 432：As shown in Fig. 10, the image of the velocity field along the Y-axis of the workshop indicates slow air movement near the walls, caused by natural convection due to temperature differences. This convection leads to convective heat transfer within the workshop's air, resulting in a reduced vertical temperature gradient.

**Fig. 10** The streamline field of section Y-axis of the workshop at 12:00

**1.13 In section 5, list the conclusions as bullet points.**

Reply：

**5.Conclusion**

**2.1 The novelty of this study needs to be clarified. Please describe this adequately.**

Reply：Modify the content in the abstract to highlight the novelty of the research.

It's on line 16：Empirical formulas only work in specific conditions due to factors like the environment, climate, and fish types,resulting in inaccurate equipment selection outcomes. Recognizing this limitation, this paper proposes to apply CFD simulation of the temperature field to accurately calculate the heat exchange value between indoor air and water, thereby predicting the heat exchange values during aquaculture activities in the aquaculture workshop.

**2.2 The validation part needs to be clarified. In Figure 2, it is unclear how to get the experimental data.**

Reply：Details of the experimental data acquisition in Figure 2 have been refined, and images of the experimental setup have been presented.

It's on line 303：In this study, temperature control equipment LogTag TRIX-8, as shown in Fig.4, was placed 15 meters southwest of the workshop, recording the outdoor temperature changes over a 24-hour period at 15-minute intervals. The collected data were fitted using a third-order Fourier's law function in MATLAB, simplifying it to a 24-hour periodic simple harmonic motion [3]. As shown in Fig.5, the outdoor temperature parameter fitting curve for October 20, 2022, has a root-mean-square error (RMSE) of 0.6302.

**Fig. 4** LogTag TRIX-8 Temperature and Humidity Sensor

**2.3 The description of the experimental part is not elaborately described.**

Reply：A detailed description of the distribution of the equipment used in the experiment has been provided

It's on line 358：a central test point is established in the center of the aquaculture room. Based on this, test points are uniformly arranged along the north-south axis at intervals of 8 meters, and along the east-west axis at intervals of 9 meters, together forming a "cross" shaped layout. Vertically, in the workshop's corridors and equipment areas, three sensors are layered at heights of 0.2m, 1.5m, and 3m. Additionally, two sensors are also layered vertically above the water pool, at heights of 1.5m and 3m, respectively. In total, 39 temperature monitoring points are set up. The arrangement is shown in Fig.5.

**2.4 he section and subsection fronts need to be distinguished.**

Reply：I have made the necessary distinctions between the section and subsection fronts in the document

**2.5 Provide proper reference for formulas 1 and 2.**

Reply：I have already added reference(s) for equations 1-2

It's on line 200：Reference is “Wang Fujun. Computational Fluid Dynamics Analysis—Principles and Applications of CFD Software [M]. Beijing. Tsinghua University Press. 2004”

**2.6 Describe elaborately how the simulation results help control water temperature equipment. They need to be more apparent to the audience.**

Reply：The numerical simulation results are not intended to assist in controlling the temperature of the equipment. Instead, they are used to predict the power required by the equipment based on the results. I have provided a more detailed description of this aspect in the Section 4.2.

It's on line 445：Through simulation and prediction, the air temperature involved in heat exchange with the water body during normal operation of the workshop was determined. This outcome can be used in the initial stages of equipment selection for the workshop, to calculate the heat exchange results after the installation of equipment. Hence, it is evident that CFD plays an irreplaceable role in the selection of equipment. Through the results of the simulation, it is known that the overall temperature field in the breeding workshop is relatively uniform

**2.7 Where is the phase change mode working, and what is the interfacial condition for the phase change?**

Reply：The phase change model plays a role in the source term of the energy equation for the temperature field, and the applicable conditions for its empirical formula are provided.

the heat absorbed by the water body during phase change is calculated by considering the change in enthalpy values associated with evaporation. This heat is then incorporated into the energy equation as a source term, allowing for a more accurate simulation of temperature field variations.

One of the empirical formulas is particularly suitable for scenarios where the flow speed is less than 0.5 m/s, aligning well with the parameters of this study.

**2.8 What is the flow governing parameters? If it is Reynolds number, then describe how to influence the Re of the flow and temperature pattern.**

Reply：The Reynolds number influences the flow, and calculations of the Reynolds number have been included in the article.

The fluid flow is slow, as measured by a hot-wire anemometer, indicating a near-wall air velocity of 0.01 m/s. Using the height of the workshop as the characteristic length, the calculated Reynolds number is 2000

**2.9 Is it force convection or mixed convection? Describe clearly.**

Reply：In the document, mixed convection is considered, and a more detailed introduction to mixed convection has been added to the article.

which leads to an inconsistent distribution of air density indoors, natural convection of indoor air is easily induced in the workshop

Therefore, by considering the effects of air density and gravity due to temperature changes, and employing the Boussinesq assumption, the buoyancy induced by temperature differences is simplified and incorporated into the source term of the energy equation.

**2.10 The description of the CFD part needs to be better. The CFD study needs to be clarified here. The authors should elaborate on this and correlate the necessity of this CFD study**

Reply：A detailed clarification of the purpose of the CFD study has been provided, aiming to predict the heat transfer situation in the workshop during normal operation and expressing its necessity in equipment selection，And details of the material parameters were added in the CFD settings for a clearer description.

Through simulation and prediction, the air temperature involved in heat exchange with the water body during normal operation of the workshop was determined. This outcome can be used in the initial stages of equipment selection for the workshop, to calculate the heat exchange results after the installation of equipment. Hence, it is evident that CFD plays an irreplaceable role in the selection of equipment. Through the results of the simulation, it is known that the overall temperature field in the breeding workshop is relatively uniform

Table 2 Material properties setting table

| project | air | water | concrete | plane skylight |
| --- | --- | --- | --- | --- |
| density（kg/m^3^） | Ideal gas  Density varies with temperature | 1000 | 2100 | 1500 |
| specific heat（J/kg$\cdot$ k） | 1006.43 | 4200 | 880 | 1050 |
| heat conductivity coefficient  （W/m$\cdot$ k） | 0.242 | 0.599 | 1.4 | 0.158 |
| absorptivity | 0 | 0.1 | 0.6 | 0.1 |
| scattering coefficient | 0 | 0 | 1 | 0 |
| diffusion coefficient | 1 | 1 | 1 | 1 |
| emissivity | 0.86 | 0.85 | 0.71 | 0.85 |

Minor issues to be modified as required

**3.1 The title would be better if the author rewrite Title. The word CFD methods and A numerical simulation both are similar.**

Reply：Appropriate adjustments have been made based on your suggestion. You pointed out that the phrases 'CFD methods' and 'A numerical simulation' are similar. Therefore, we can consider removing redundant or similar expressions. The revised title could be:Optimizing Heat Pump Unit Selection for Recirculating Aquaculture Workshops through Computational Fluid Dynamics

**3.2 In Abstract, the problem statement is articulated well, identifying the specific issue or gap that the research aims to address. It would be beneficial to clearly define the numerical methods for solving the computational domain. It is recommended to provide a bit more detail on the key results or outcomes, ensuring that readers get a glimpse of the research's contribution.**

Reply：I have added more details and results to the abstract of the article, with the modifications as follows:Comparison with experimental data reveals a root mean square error of 0.45°C for the simulated results. During summer, the highest cooling load occurs at 16:00, reaching 94.6 kW. It is recommended to employ the Daikin GCHP-40MAH ground source heat pump as the water temperature control equipment. CFD simulation validates its effectiveness in shaping the indoor temperature field post-installation.

**3.3 The researcher has effectively outlined the objective and scope of the study. However, the review of existing literature appears to be insufficient, and there is a notable gap in addressing the current state of knowledge in the field of fluid-solidcoupling simulation method to describe the energy transfer process analysis. The author should expand the literature review section to encompass a more comprehensive overview of recent relevant studies, theories, and methodologies related to the research topic.**

Reply：References related to recent years are added:

Shenghan Zhou and colleagues conducted a study using FLUENT software to simulate the temperature field in a plant factory. Their research focused on the detailed three-dimensional modeling of temperature distribution influenced by LED light heat dissipation and boundary conditions, emphasizing the significance of accurate modeling in such environments[1]. Long Yang and his team utilized FLUENT in their research to simulate the air flow and temperature fields in a gosling house with vertical wall attached jet ventilation in a cold region. This study investigated the effects of different air supply velocities on the thermal environment, providing essential insights for managing ventilation in livestock housing under cold climate conditions[2]. Cruz Ernesto Aguilar Rodriguez and collaborators applied FLUENT software in their research for simulating heat and mass transfer processes in greenhouses. Their work highlighted the importance of heating systems like pipe heating and its impact on crop yields, energy consumption, and operational costs[3]. The study employed computational fluid dynamics to analyze factors such as air movement, temperature gradients, and crop transpiration, crucial for understanding the dynamics of greenhouse systems. The aforementioned studies demonstrate that CFD has been widely applied in the agricultural field, and the use of Fluent software for temperature field simulations is quite mature.

**3.4 In section 2.1 detail of Physical Model of the Workshop Space, page 4, the description of designing workshop space with boundary condition is not clear. It would be very helpful for the readers if the author provides a Figure of physical model with detailed dimensions and boundary conditions of workshop space.**

Reply：Added a physical model diagram with detailed dimensions and a real shot of the workshop

**Fig. 1-1** Architectural diagram of the recirculating aquaculture workshop

**Fig.1-2** Main view of the recirculating aquaculture workshop

**Fig.1-3** Top view of the recirculating aquaculture workshop

**3.5 In section Meshing and validation, page 7, Did the authors use the block mesh and prism layer mesh for the refinement of the investigated area inside the workshop? As we know, the prism layer mesh is used with a core volume mesh to generate orthogonal prismatic cells next to wall boundaries. This layer of the cell is necessary to improve the accuracy of the flow solution. Moreover, I have not found any information regarding Grid independence test for mesh validation in this manuscript regarding this issue.**

Reply："The entire workshop is quite orderly. In the mesh division, block meshes were used, and an expansion layer was set at the position where the air computational domain contacts the wall. The paper has changed the image to a cross-sectional mesh diagram for a more accurate representation of the mesh situation, and added a line graph of experimental data for the grid independence test."

**Fig. 2** Schematic diagram of the grid division for the computational domains of the second scheme

**Fig. 3** Grid Independence Line Chart

**3.6 It mentions the use of the Finite Volume Method (FVM) but does not describe the methodology or approach in any detail. The mathematical treatment for unsteady simulation part is also not lucid. Please check and modify whatever is required.**

Reply：The text provides a detailed introduction to the methodology of FVM when it is mentioned

The finite volume method was adopted to discretize the model equations, divide the computational domain for the numerical simulation into a grid, ensuring that there is a unique control volume around each grid point. Obtain discrete equations by integrating the differential equations

And explained the mathematical approach of the semi-implicit scheme used for the discretization of the unsteady terms.

A semi-implicit format was chosen for time discretization, to reduce the discretization error caused by time discretization by obtaining the average of explicit and implicit schemes.

**3.7 In Section 2.3, the authors defined the state of indoor air can be regarded as three-dimensional, unsteady-state, incompressible Newtonian fluid motion, and equation 1 are not consistent with the mentioned references [10-14 and 15-17], like notations of subscripts and Boussinesq assumption are not appropriately used. Please check and correct me if I am wrong. Moreover, all vector natation’s and variables declarations are not adequate, which, lowers the scientific quality of the paper. Please check and modify whatever is required.**

Reply：Further explanations of the symbols in Equation 1 were provided, and the role of Boussinesq approximation in natural convection was elaborated in more detail in the subsequent text

**3.8Figures provides valuable insights into the paper. However, a comprehensive evaluation suggests that there are areas to improve from Figure 2 to Figure 5. The title of Figures must be consistent with the results and level of Figures heading. Please correct all Figures. For example, “Fig. 4 Error Distribution Diagram between Simulated and Measured Values” is not consistent with the level of Fig 4.**

Reply：Some adjustments have been made for this problem

**3.9 It would be appreciated if the authors include the validation of the numerical results with published results.**

Reply：The paper does not contain content comparing it with other articles, but the sample size of the experiment was increased to verify the accuracy of the model.

**3.10 There are many typographic mistakes in the text and those should be corrected**

Reply：Many details were found and adjusted

**3.11 The formatting in this manuscript and format of all references in this manuscript does not meet the requirement of this journal. Please see and follow the guideline for writing the manuscript of this journal.**

Reply：Fill and adjust the reference format

**4.1 Writing of introduction should be improved as the sequence of keywords are not followed for potential readers.**

Reply：Combining the introduction and the sequence of presentation in the article, the keywords have been rearranged to better align with the requirements of the paper.

**Keywords:** Aquaculture workshop; Computational Fluid Dynamics; Temperature field simulation; Water temperature regulation equipment; Equipment selection

**4.2 References are not called properly and references [1-5] are cited many times in many places which can be handled properly.**

Reply：Delete the error reference

**4.3 Basic control equations requires more details specially how the radiation affects the flow and in which condition this should be addressed.**

Reply：Added detailed descriptions of the basic control equations, and explained how energy changes due to phase transitions, radiation, etc., further affect the flow field changes inside the workshop, achieving coupling.、

When in the equation, it is the continuity equation; when, it is the momentum equation; and when, it is the energy equation.When phenomena such as phase change, radiation, and convection occur, causing temperature changes, the air density changes accordingly, which then affects the momentum equation and influences air flow, realizing multi-model coupling.

**4.4 The numerical uncertainty due to the weather condition, i.e. sunny or cloudy, needs to be addressed. Only one day data for producing the results are not convincing.**

Reply：The objective of this study is to select temperature control equipment, requiring predictions for the building's environment under extreme weather conditions, specifically data under the hottest conditions in summer. Therefore, temperature situations under cloudy and rainy conditions were not considered. The paper has been updated to state that the experiment lasted for three days. Despite a slight decrease in simulation accuracy, it still meets the requirements for simulation precision.

**4.5 Calculation method has not written with adequate information. The Blackbox of the commercial software FLUENT has not opened and discussed in the manuscript.**

Reply：The settings for the material properties were displayed in accordance with the requirements of Fluent's configuration.

Table 2 Material properties setting table

| project | air | water | concrete | plane skylight |
| --- | --- | --- | --- | --- |
| density（kg/m^3^） | Ideal gas  Density varies with temperature | 1000 | 2100 | 1500 |
| specific heat（J/kg$\cdot$ k） | 1006.43 | 4200 | 880 | 1050 |
| heat conductivity coefficient  （W/m$\cdot$ k） | 0.242 | 0.599 | 1.4 | 0.158 |
| absorptivity | 0 | 0.1 | 0.6 | 0.1 |
| scattering coefficient | 0 | 0 | 1 | 0 |
| diffusion coefficient | 1 | 1 | 1 | 1 |
| emissivity | 0.86 | 0.85 | 0.71 | 0.85 |

**4.6 The given simulation results are not convincing to justify the need of CFD study for this type of workshop. Temperature variation are within 2-3 degree Celsius. Placing thermal sensors for few places are adequate to read temperature data for several days or so. Thereafter, Machine Learning Approach (MLA) can be utilized to predict the time of maximum cooling load. What the significance of study CFD that supersede the modern MLA approach.**

Reply：Sorry, teacher, I didn't make it clear in the article that the premise of placing sensors in the workshop to read temperatures is assuming that the equipment selection has already been completed. CFD can simulate the temperature distribution in the workshop without finalizing the equipment selection, which is crucial for evaluating the impact of different equipment selections on temperature distribution. Additionally, I am considering combining the MLA method with numerical simulations to more rapidly predict the occurrence time of the maximum cooling load. Below is my revision to the article to better articulate the role of CFD in equipment selection.

I included the sentence "thereby predicting the heat exchange values during aquaculture activities in the aquaculture workshop" in the abstract to provide a clearer representation of the role of CFD in its equipment selection.
