# Supplementary material for: Optimizing heat pump unit selection for recirculating aquaculture workshops through computational fluid dynamics
Source: PLoS One. 2024 Jul 10;19(7):e0299997. doi: 10.1371/journal.pone.0299997 (PMC11236187; doi:10.1371/journal.pone.0299997)
Supplement: Attachment — Submitted filename: REVIEWER COMMENTS.docx [file pone.0299997.s004.docx]

Selection of Heat Pump Units for Recirculating Aquaculture Workshops Using Computational Fluid Dynamics: Numerical Simulation

Manuscript Number: PONE-D-23-39332:

The following comments must be taken into consideration before publishing:

1. In the abstract, the authors must avoid general description and focusing on the methodology, the results, and conclusions.
2. In section 1. Introduction, the authors spent more than on page talking about the well-known information about CFD role for solving heat transfer model. This introduction must be reduced and the introduction should focus on the main paper objective. And more references must be added about the objective of the paper. Moreover, nothing mentioned about the CFD software that has been used by the authors.
3. In section 2.1, a schematic diagram for the physical model must be added to demonstrate the details that were mentioned in this section.
4. In section 2.2 Computational Domain and Grid Division, lines 175-179, the choosing of scheme 2 must be justified by drawing a graph between the values of highest temperature of the indoor air and the number of the Grids for each scheme.
5. It preferable to present a cross-section for the Grid mesh of Fig.1.
6. Add reference(s) for the equations 1-2.
7. In section 2.3 Basic Control Equations, lines-206-207, how did the authors decide that the flow is laminar? Justify the assumption by calculating Rayleigh number.
8. The range of application of Equation 3.
9. In section 2.5 Computational Domain and Boundary Conditions, The author must add the mathematical formulation of the boundary conditions mentioned in this section.
10. The details of the equation 5. Where did the authors get this equation?
11. In section 4.2, The Figure 5 should be Figure 6.
12. In section 4.2, a streamlines of velocity distribution.
13. In section 5, list the conclusions as bullet points.
